# Supplementary material for: Australian University Nursing and Allied Health Students’ and Staff Physical Activity Promotion Preparedness and Knowledge: A Pre-Post Study Using an Educational Intervention
Source: Int J Environ Res Public Health. 2022 Jul 28;19(15):9255. doi: 10.3390/ijerph19159255 (PMC9367832; doi:10.3390/ijerph19159255)
Supplement: Supplementary file 1 [file ijerph-19-09255-s001.zip › ijerph-1779141-supplementary.pdf]

SUPPLEMENTARY MATERIALS

File S1 Student survey

Physical Activity Promotion by health professionals

One in two Australian adults are not meeting the national physical activity guidelines. Insufficient physical activity is a major risk factor for chronic disease and death, and it is estimated to cost Australia $805 million annually. Physical activity promotion by health professionals is viewed as a key strategy to increase physical activity levels. Considering this, we would like to investigate physical activity promotion by health professionals within the Faculty of Health at the University of Canberra to gain an understanding of current education and practice to inform future teaching and clinical practice.

This survey will take less than 10 minutes and is completely voluntary, anonymous and confidential.

Research Team

Nicole Freene (Physiotherapy), Mark Naunton (Pharmacy), Ann Gates (ExerciseWorks, UK), Andrew Flood (Psychology), Reza Mortazavi (Diagnostic Pathology), Jaqi Bousie (Physiotherapy), Milli Blenkin (Counselling), Lynn Cheong (Pharmacy), Madeleine Shanahan (Medical Imaging), Myra Leung (Vision Science & Optometry), Steve Isbel (Occupational Therapy), Ben Rattray (Sport & Exercise Science), Nick Ball (Sport & Exercise Science), Michelle Minehan (Nutrition & Dietetics), Kasia Bail (Nursing), Sally De-Vitry Smith (Midwifery).

Project Aim

The aim of this survey is to investigate physical activity promotion by health professionals within Faculty of Health students at the University of Canberra.

Are you currently a student within the Faculty of Health, University of Canberra and planning on a career as a health professional?

- o Yes
- o No – Thank you for your time. Unfortunately, to be included in this study you must be currently a student within the Faculty of Health, University of Canberra.

Benefits of the Project

Findings from this study will inform the training and practice of health professionals, preparing our future health workforce to take advantage of opportunities, and improve the health and well-being of our patients.

Participant Involvement, Confidentiality and Anonymity

UC Faculty of Health students who agree to participate in the research are asked to complete this online survey, which should only take about 10 minutes of your time.

Participation in the research is completely voluntary and UC Faculty of Health students may, without any penalty, decline to take part or withdraw their response at any time without providing an explanation, or refuse to answer a question.

Your answers will be completely anonymous and confidential. The only potential risks to participation relate to privacy and confidentiality. Please be assured that all the data collected from UC Faculty of Health students will be stored securely and only accessed by the research team.

The research outcomes may be presented at conferences and written up for publication. However, in all these reports, the privacy and confidentiality of individuals will be protected.

Data Storage

The information collected will be stored securely on a password protected computer throughout the project and then stored at the University of Canberra for the required five year period after which it will be destroyed according to university protocols.

Ethics Committee Clearance

The project has been approved by the Human Research Ethics Committee of the University (HREC – 4568).

Queries and Concerns

Queries or concerns regarding the research can be directed to Dr Nicole Freene, phone 02 6201 5550 or email nicole.freene@canberra.edu.au .

A summary of the research report can be forwarded to you when published. If you would like to receive a copy of the report once completed, please email Nicole Freene nicole.freene@canberra.edu.au

If you have any complaints or reservations about the ethical conduct of this research, you may contact the University of Canberra's Research Ethics & Integrity Unit team via telephone 02 6206 3916 or email humanethicscommittee@canberra.edu.au or researchethicsandintegrity@canberra.edu.au

If you would like some guidance on the questions you could ask about your participation please refer to the Participants' Guide located at http://www.canberra.edu.au/ucresearch/attachments/pdf/a-m/Agreeing-to-participate-in-research.pdf

If you decide to continue, you are consenting to taking part in the survey.

Thank you for taking the time to complete this survey ☺

Physical Activity Promotion by health professionals

Physical Activity: includes any activity from a low intensity level, such as walking or gardening, to a high intensity level, such as playing a competitive sport.

1. To what extent do you agree or disagree with the following statements: ***(please select only one for each statement)***

	**Strongly Agree**	**Agree**	**Not Sure**	**Disagree**	**Strongly Disagree**
a. Taking the stairs at work and generally being more active each day is enough physical activity to improve health	1	2	3	4	5
b. Half an hour of walking on most days is all the physical activity that is needed for good health	1	2	3	4	5
c. Physical activity that is good for health must make you puff and pant	1	2	3	4	5
d. Several short walks on most days is better than one round of golf per week for good health	1	2	3	4	5
e. Discussing the benefits of a physically active lifestyle with patients/individuals is part of the health professional's role	1	2	3	4	5
f. Suggesting to patients/individuals ways to increase daily physical activity is part of the health professional's role	1	2	3	4	5
g. I would feel confident in giving general advice to patients/individuals on a physically active lifestyle	1	2	3	4	5

h. I would feel confident in suggesting specific physical activity programs for my patients/individuals	1	2	3	4	5
i. Health professionals should be physically active to act as a role model for their patients/individuals	1	2	3	4	5

2. How often do you think the following would prevent clinicians in your discipline from promoting a physically active lifestyle to their patients/individuals? ***(please select only one for each statement)***

	**Never**	**Rarely**	**Sometimes**	**Often**	**Very Often**
a. Lack of time	1	2	3	4	5
b. Lack of counselling skills	1	2	3	4	5
c. Lack of remuneration for promoting physical activity	1	2	3	4	5
d. Lack of interest in promoting physical activity	1	2	3	4	5
e. Feeling it would not change the patient's/individuals behaviour	1	2	3	4	5
f. Feeling it would not be beneficial for the patient/individual	1	2	3	4	5
g. Other (Please specify)	1	2	3	4	5

3. What kinds of physical activity promotion (beyond therapeutic exercise) would be feasible for clinicians in your discipline to deliver to their patients/individuals? ***(please select only one for each statement)***

	**Highly Feasible**	**Somewhat Feasible**	**Not Sure**	**Not Really Feasible**	**Totally Unfeasible**

a. Brief counselling integrated into regular consultations	1	2	3	4	5
b. Separate one-on-one consultations	1	2	3	4	5
c. Group sessions	1	2	3	4	5
d. Distribution of resources (e.g. brochures)	1	2	3	4	5

4. Some general questions about you:

a. Your gender: ○ Female ○ Male ○ Other

b. Your age in years:

c. In which discipline do you study?

○ Occupational Therapy
○ Physiotherapy
○ Pharmacy
○ Nutrition & Dietetics
○ Nursing
○ Midwifery
○ Psychology
○ Vision Science & Optometry
○ Speech Pathology
○ Counselling
○ Sport & Exercise Science
○ Medical Radiation Science
○ Public Health
○ Diagnostic Pathology

d. Please indicate if you are enrolled in an undergraduate or postgraduate degree?

○ Undergraduate (Bachelor)
○ Postgraduate (Master)

e. What year are you in within your degree?

○ First
○ Second
○ Third
○ Fourth

5a. Are you aware of the National Physical Activity and Sedentary Behaviour Recommendation for Australian Adults?

○ Yes

○ No

b. If yes, please describe the National Physical Activity and Sedentary Behaviour Recommendation for Australian Adults here

7. Finally, about your own physical activity: How physically active do you think you are currently compared with other Australians of your sex and age? *(please select one)*

○ Much more active
○ Slightly more active
○ About the same
○ Slightly less active
○ Much less active

Thank you for completing the survey!

File S2 Staff survey

UNIVERSITY OF CANBERRA
AUSTRALIA'S CAPITAL UNIVERSITY

Physical Activity Promotion by health professionals

One in two Australian adults are not meeting the national physical activity guidelines. Insufficient physical activity is a major risk factor for chronic disease and death, and it is estimated to cost Australia $805 million annually. Physical activity promotion by health professionals is viewed as a key strategy to increase physical activity levels. Considering this, we would like to investigate physical activity promotion by health professionals within the Faculty of Health at the University of Canberra to gain an understanding of current education and practice to inform future teaching and clinical practice.

This survey will take less than 10 minutes and is completely voluntary, anonymous and confidential.

Research Team

Nicole Freene (Physiotherapy), Mark Naunton (Pharmacy), Ann Gates (ExerciseWorks, UK), Andrew Flood (Psychology), Reza Mortazavi (Diagnostic Pathology), Jaqi Bousie (Physiotherapy), Milli Blenkin (Counselling), Lynn Cheong (Pharmacy), Madeleine Shanahan (Medical Imaging), Myra Leung (Vision Science & Optometry), Steve Isbel (Occupational Therapy), Ben Rattray (Sport & Exercise Science), Nick Ball (Sport & Exercise Science), Michelle Minehan (Nutrition & Dietetics), Kasia Bail (Nursing), Sally De-Vitry Smith (Midwifery).

Project Aim

The aim of this survey is to investigate physical activity promotion by health professionals within Faculty of Health staff at the University of Canberra.

Are you currently employed as a staff member within the Faculty of Health, University of Canberra?

- ○ Yes
- ○ No – Thank you for your time. Unfortunately, to be included in this study you must be currently working as a staff member within the Faculty of Health, University of Canberra.

Benefits of the Project

Findings from this study will inform the training and practice of health professionals, preparing our future health workforce to take advantage of opportunities, and improve the health and well-being of our patients.

Participant Involvement, Confidentiality and Anonymity

UC Faculty of Health staff members who agree to participate in the research are asked to complete this online survey, which should only take about 10 minutes of your time.

Participation in the research is completely voluntary and UC Faculty of Health staff members may, without any penalty, decline to take part or withdraw their response at any time without providing an explanation, or refuse to answer a question.

Your answers will be completely anonymous and confidential. The only potential risks to participation relate to privacy and confidentiality. Please be assured that all the data collected from UC Faculty of Health staff members will be stored securely and only accessed by the research team.

The research outcomes may be presented at conferences and written up for publication. However, in all these reports, the privacy and confidentiality of individuals will be protected.

Data Storage

The information collected will be stored securely on a password protected computer throughout the project and then stored at the University of Canberra for the required five year period after which it will be destroyed according to university protocols.

Ethics Committee Clearance

The project has been approved by the Human Research Ethics Committee of the University (HREC – 4568).

Queries and Concerns

Queries or concerns regarding the research can be directed to Dr Nicole Freene, phone 02 6201 5550 or email nicole.freene@canberra.edu.au .

A summary of the research report can be forwarded to you when published. If you would like to receive a copy of the report once completed, please email Nicole Freene nicole.freene@canberra.edu.au

If you have any complaints or reservations about the ethical conduct of this research, you may contact the University of Canberra's Research Ethics & Integrity Unit team via telephone 02 6206 3916 or email humanethicscommittee@canberra.edu.au or researchethicsandintegrity@canberra.edu.au

If you would like some guidance on the questions you could ask about your participation please refer to the Participants' Guide located at http://www.canberra.edu.au/ucresearch/attachments/pdf/a-m/Agreeing-to-participate-in-research.pdf

If you decide to continue, you are consenting to taking part in the survey.

Thank you for taking the time to complete this survey ☺

Physical Activity in Nursing & Allied Health Professional Practice

Physical Activity: includes any activity from a low intensity level, such as walking or gardening, to a high intensity level, such as playing a competitive sport.

1. To what extent do you agree or disagree with the following statements: ***(please select only one for each statement)***

	**Strongly Agree**	**Agree**	**Not Sure**	**Disagree**	**Strongly Disagree**
a. Taking the stairs at work and generally being more active each day is enough physical activity to improve health	1	2	3	4	5
b. Half an hour of walking on most days is all the physical activity that is needed for good health	1	2	3	4	5
c. Physical activity that is good for health must make you puff and pant	1	2	3	4	5
d. Several short walks on most days is better than one round of golf per week for good health	1	2	3	4	5
e. Discussing the benefits of a physically active lifestyle with patients/individuals is part of the health professional's role	1	2	3	4	5
f. Suggesting to patients/individuals ways to increase daily physical activity is part of the health professional's role	1	2	3	4	5

g. I feel confident in giving general advice to patients/individuals on a physically active lifestyle	1	2	3	4	5	N/A
h. I feel confident in suggesting specific physical activity programs for my patients/individuals	1	2		4	5	N/A
i. Health professionals should be physically active to act as a role model for their patients/individuals	1	2	3	4	5	

2. How often do you think the following would prevent clinicians in your discipline from promoting a physically active lifestyle to their patients/individuals? ***(please select only one for each statement)***

	Never	Rarely	Sometimes	Often	Very Often
a. Lack of time	1	2	3	4	5
b. Lack of counselling skills	1	2	3	4	5
c. Lack of remuneration for promoting physical activity	1	2	3	4	5
d. Lack of interest in promoting physical activity	1	2	3	4	5
e. Feeling it would not change the patient's/individuals behaviour	1	2	3	4	5
f. Feeling it would not be beneficial for the patient/individual	1	2	3	4	5
g. Other (Please specify)	1	2	3	4	5

3. What kinds of physical activity promotion (beyond therapeutic exercise) would be feasible for clinicians in your discipline to deliver to their patients/individuals? ***(please select only one for each statement)***

	Highly Feasible	Somewhat Feasible	Not Sure	Not Really Feasible	Totally Unfeasible
a. Brief counselling integrated into regular consultations	1	2	3	4	5
b. Separate one-on-one consultations	1	2	3	4	5
c. Group sessions	1	2	3	4	5
d. Distribution of resources (e.g. brochures)	1	2	3	4	5

4. Some general questions about you:

a. Your gender: ○ Female ○ Male ○ Other

b. Your age in years: ○ <35 ○ 35-44 ○ 45-54 ○ >54

c. In which discipline do you work?

○ Occupational Therapy
○ Physiotherapy
○ Pharmacy
○ Nutrition & Dietetics
○ Nursing
○ Midwifery
○ Psychology
○ Vision Science & Optometry
○ Speech Pathology
○ Counselling
○ Sport & Exercise Science
○ Medical Radiation Science
○ Public Health
○ Diagnostic Pathology

d. Are you a trained health professional? ○ Yes ○ No

e. If yes, how many years is it since you completed your first degree in health?

5. Are you currently practicing clinically as a health professional?

- ○ Yes – continue to questions below
- ○ No – skip to question 6

a. How often do you encourage your patients to have a more physically active lifestyle (beyond therapeutic exercise) in the last month? *(please select one)*

○ Never

○ Rarely, only 1 or 2 patients

○ Sometimes, perhaps 3-5 patients

○ Often, perhaps 6 -9 patients

○ More often, 10 or more patients

b. In what kind of practice do you work?

○ Private practice

○ Community Health

○ Public hospital

○ Private hospital

○ Other __________________________

c. Average number of patients you see each week:

d. Your number of years in practice:

e. Usual number of hours worked each week:

6a. Are you aware of the National Physical Activity and Sedentary Behaviour Recommendation for Australian Adults?

○ Yes

○ No

b. If yes, please describe the National Physical Activity and Sedentary Behaviour Recommendation for Australian Adults here

7. Finally, about your own physical activity: How physically active do you think you are currently compared with other Australians of your sex and age? ***(please select one)***

○ Much more active
○ Slightly more active
○ About the same
○ Slightly less active
○ Much less active

Thank you for completing the survey!

File S3 Curriculum audit tool

	A	B	C	D	E	F	G	H	I	J	K	L	M	N	O	P	Q	R	S	T	U	V
1	**Discipline -**																					
2																						
3	**""Note: specfic PA content refers to Aust PA guidelines from early yrs to older adults eg: adults -MVPA 150-300 mins/wk, strength x2/wk, reduce SB""**																					
4	**(non-treatment PA rather than treatment or theraputic exercise)**																					
5	https://www1.health.gov.au/internet/main/publishing.nsf/Content/health-pubhlth-strateg-phys-act-guidelines																					
6																						
7	**Unit number**	**Unit name**	**Unit convenor**	**Year of degree**	**Semester delivered (1=S1, 2=S2, 3=WS, 4=other)**	**Average number of students enrolled** (based on last semester)	**Inter-professional unit (1=Y, 2=N)** ie: students from various disciplines	**Specific PA content included (1=Y, 2=N)**	**Specific PA content included in lectures (1=Y, 2=N)**	**Number of lectures including specific PA content**	**Hours allocated to specific PA content in lectures**	**Specific PA content included in tutorials (1=Y, 2=N)**	**Number of tutorials including specific PA content**	**Hours allocated to specific PA content in tutorials**	**Assessment of specifc PA content (1=Y, 2=N)**	**Assessment details (free text)** eg: theory exam, practical exams (brief intervention), written assignment. Can include weighting if applicable/available	**PA & SB guidelines (FITT) outlined (1=Y, 2=N)**	**Comments**	**Health benefits of PA outlined (1=Y, 2=N)**	**Comments** eg: specific NCDs and diagnostic groups targeted	**PA promotion (including Ax, counselling, etc) outlined (1=Y, 2=N)**	**Comments** eg: inclusion of barriers, enbalers and issues such as inequity
8																						
9																						
10																						

Table S1. Example descriptions of the Australian physical activity and sedentary behaviour guidelines for adults and scoring.

Participant	Description	Score
Student	be active most days to a weekly total of 2.5-5 hours of moderate exercise	D+I
	Regular physical activity	-
	Dpt Health Report on the recommendations and guidelines for how much physical and sleep is needed to prevent the onset of illness in children and adults. Relates a sedentary lifestyle to particular health risk factors and conditions.	-
	2.5-5 hours moderate physical activity per week, strength training at least 2x per week, limit total sitting time and break up long periods of sitting as often as possible	D+I+R+SB
	at least thirty minutes a day of moderate exercise, i.e brisk walk, able to talk but not able to sing.	D+I
	30 minutes per day of moderate to vigorous exercise, at least 5 days per week. Minimise sitting and break up long periods of sitting with a walk.	D+I+SB
Staff	Act as guidelines for healthy amount of mod and vig physical activity and sitting time for Australian adults. Recommendation is for 30min of physical activity a day, including both moderate and vigorous intensity.	D+I
	Do more, sit less	-
	Be active most days, include moderate activity (walking etc 2.5-5hrs), or vigorous activity (jogging etc 1.5-2.5hrs), add muscle strengthening 2 days/wk (push ups etc)	D+I+R
	Adults, active most days per week, 150-300 mins moderate intensity or 75-150 mins vigorous intensity exercise or equivalent combination, at least 2 days strength exercise, limit sitting	D+I+R+SB
	To be active most days moderately and vigorous activity at least once a week	I

D: duration; I: intensity; R: resistance/strength; SB: sedentary behaviour/sitting

Table S2. Health students and staff Awareness and Knowledge of the Australian Physical Activity and Sedentary Behaviour guidelines.

Participants	Awareness, yes n (%)		Duration, correct n (%)		Intensity, correct n (%)		Resistance training, correct n (%)		Sedentary Behaviour, correct n (%)		Complete guidelines, correct n (%)	
	T1	T2	T1	T2	T1	T2	T1	T2	T1	T2	T1	T2
Student (T1 n=267, T2 n=329)	124 (46.4)	174 (52.9)	84 (31.5)	106 (32.2)	70 (26.2)	95 (28.9)	30 (11.2)	40 (12.2)	21 (7.9)	31 (9.4)	7 (2.6)	20 (6.1)
Staff (T1 n=38, T2 n=30)	18 (47.4)	22 (73.3)	11 (28.9)	12 (40)	11 (28.9)	15 (50)	3 (7.9)	6 (20)	4 (10.5)	7 (23.3)	2 (5.3)	3 (10)

T1: 2020; T2: 2021

Video S1. Physical activity promotion by health professionals: Educating our future workforce https://www.youtube.com/watch?v=69JbcoW0tbc
